# Association of the classification of intraoperative adverse events (ClassIntra) with complications and neurological outcome after neurosurgical procedures: a prospective cohort study

**DOI:** 10.1007/s00701-023-05672-w

**Published:** 2023-07-05

**Authors:** Richard Drexler, Franz L. Ricklefs, Tobias Pantel, Jennifer Göttsche, Rainer Nitzschke, Christian Zöllner, Manfred Westphal, Lasse Dührsen

**Affiliations:** 1 Department of Neurosurgery, University Medical Center Hamburg-Eppendorf, Hamburg, Germany; 2 Department of Anesthesiology, University Medical Center Hamburg-Eppendorf, Hamburg, Germany

**Keywords:** Neurosurgery, Craniotomy, Outcome, Intraoperative, Risk, Complication

## Abstract

**Purpose:**

To analyze the reliability of the classification of intraoperative adverse events (ClassIntra) to reflect intraoperative complications of neurosurgical procedures and the potential to predict the postoperative outcome including the neurological performance. The ClassIntra classification was recently introduced and found to be reliable for assessing intraoperative adverse events and predicting postoperative complications across different surgical disciplines. Nevertheless, its potential role for neurosurgical procedures remains elusive.

**Methods:**

This is a prospective, monocentric cohort study assessing the ClassIntra in 422 adult patients who underwent a neurosurgical procedure and were hospitalized between July 1, 2021, to December 31, 2021. The primary outcome was the occurrence of intraoperative complications graded according to ClassIntra and the association with postoperative outcome reflected by the Clavien-Dindo classification and comprehensive complication index (CCI). The ClassIntra is defined as intraoperative adverse events as any deviation from the ideal course on a grading scale from grade 0 (no deviation) to grade V (intraoperative death) and was set at sign-out in agreement between neurosurgeon and anesthesiologist. Secondary outcomes were the neurological outcome after surgery as defined by Glasgow Coma Scale (GCS), modified Rankin scale (mRS), Neurologic Assessment in Neuro-Oncology (NANO) scale, National Institute Health of Strokes Scale (NIHSS), and Karnofsky Performance Score (KPS), and need for unscheduled brain scan.

**Results:**

Of 442 patients (mean [SD] age, 56.1 [16.2]; 235 [55.7%] women and 187 [44.3%] men) who underwent a neurosurgical procedure, 169 (40.0%) patients had an intraoperative adverse event (iAE) classified as ClassIntra I or higher. The NIHSS score at admission (OR, 1.29; 95% CI, 1.03–1.63, female gender (OR, 0.44; 95% CI, 0.23–0.84), extracranial procedures (OR, 0.17; 95% CI, 0.08–0.61), and emergency cases (OR, 2.84; 95% CI, 1.53–3.78) were independent risk factors for a more severe iAE. A ClassIntra ≥ II was associated with increased odds of postoperative complications classified as Clavien-Dindo (*p* < 0.01), neurological deterioration at discharge (*p* < 0.01), prolonged hospital (*p* < 0.01), and ICU stay (*p* < 0.01). For elective craniotomies, severity of ClassIntra was associated with the CCI (*p* < 0.01) and need for unscheduled CT or MRI scan (*p* < 0.01). The proportion of a ClassIntra ≥ II was significantly higher for emergent craniotomies (56.2%) and associated with in-hospital mortality, and an unfavorable neurological outcome (*p* < 0.01).

**Conclusion:**

Findings of this study suggest that the ClassIntra is sensitive for assessing intraoperative adverse events and sufficient to identify patients with a higher risk for developing postoperative complications after a neurosurgical procedure.

**Supplementary Information:**

The online version contains supplementary material available at 10.1007/s00701-023-05672-w.

## Introduction

Surgeons and anesthesiologists strive for the best possible outcome of their surgeries with the greatest chance for recovery of the patients. Therefore, monitoring and quality improvement is increasingly important in surgical specialties. As there are well-defined scores and classifications to describe the postoperative course regarding morbidity, mortality and neurological status [13, 20, 34], no standardized grading system for assessing intraoperative complications has been integrated into clinical practice by now. Nevertheless, the necessity for classifying adverse events during surgical procedures had been recognized in recent years and several recommendations as well as guidelines were presented [19, 21, 26, 30]. As a result, Dell-Kuster and colleagues introduced the classification of intraoperative adverse events (ClassIntra) which provided convincing results when associated with postoperative complications [11]^ (p)^. The ClassIntra classification is based on an initial description from 2015, at that time named CLASSIC and developed in a Delphi process [30]. Now, based on this, the ClassIntra has been described as a five-level classification that covers all surgical and anesthesiological events between skin incision and skin closure. The five-level classification was adapted from the widely used Clavien Dindo score for recording postoperative complications [8], and the current study by Dell-Kuster and colleagues was conducted on a cohort of 2520 patients including all surgical disciplines as well as anesthesia techniques. The ClassIntra defines intraoperative adverse events as any deviation from the ideal course on a grading scale from grade 0 (no deviation) to grade V (intraoperative death). A need for an additional intraoperative treatment or intervention due to an adverse event is defined as grade II or higher [11]^ (p)^.

Neurosurgery is a high-risk surgical specialty and occurrence of complications is closely related with transient or persistent neurological deficits impacting patients’ quality of life and health status [16, 31, 32]. Therefore, establishing quality indicators and improvement programs has been of great interest in the past decade. Recent studies focused mainly on the postoperative outcome [4, 9, 33, 37]. One study by Wong and colleagues reviewed patterns and frequencies of intraoperative adverse events in neurosurgery, and reported about a significant amount of avoidable events with optimized standards of perioperative management [43]. Even though the perioperative complications have been of great interest in the past years and recent studies have analyzed type of perioperative complications during neurosurgical intervention [6, 23, 32, 38, 42], there is no widely applied classification system in current and daily practice. Furthermore, the necessity of admission to an intensive care unit after craniotomy is a discussed topic and several criteria were defined to stratify patients according to their risk profile [2, 10]. A standardized grading system for intraoperative adverse events was not considered in these studies but is urgently needed for estimating possible complications after surgery.

Within this context, we conducted a study to assess the incidence of intraoperative adverse events graded according to the ClassIntra classification and analyzed its potential to predict the postoperative outcome after neurosurgical procedures. Specifically, we evaluated the complications after surgery with the Clavien-Dindo classification and Comprehensive Complication Index and assessed the change of patients’ neurological status between admission and discharge. We hypothesized that a more severe intraoperative adverse event would be closely related with a higher probability of postoperative complications and a worse neurological outcome.

## Methods

A prospective, monocentric cohort study was conducted to define the ClassIntra grade in patients who underwent a procedure at the Department of Neurosurgery, University Medical Center Hamburg-Eppendorf (Germany) between July 1, 2021, and December 31, 2021. The ClassIntra grade was assessed as previously described [11]^ (p)^ and the grading was set at sign-out in agreement between neurosurgeon and anesthesiologist. The ClassIntra grades were defined as following: grade 0 as no deviation from the ideal intraoperative course, grade I as any deviation without the need for additional treatment, grade II as any deviation with the need for any additional minor treatment, grade III as any deviation with the need for moderate treatment, grade IV as any deviation with the need for major and urgent treatment, and grade V as intraoperative death [11]^ (p)^. The exact definition with neurosurgical examples for each grade are listed in Table 1. All neurosurgical procedures were consecutively included in the previously mentioned period. The study was registered with ClinicalTrials.gov (NCT 04956835) and ethical approval was granted from medical ethics committee of the Hamburg chamber of physicians (2021–300,064-WF). Informed written consent was obtained from all patients. Patients’ data were collected prospectively, and various outcome scores assessed one day before surgery and at time of discharge. Patients under 18 years of age and same-day surgeries were excluded from the study.

**Table 1:** ClassIntra classification of intraoperative adverse events. The standard definitions are defined by Dell-Kuster et al. [11] and neurosurgical examples were added according to the study

Grade	Definition	Neurosurgical examples
Grade 0	No deviation from the ideal intraoperative course	-
Grade I	Any deviation from the ideal intraoperative course: • Without the need for any additional treatment or intervention • Patient with no or mild symptoms	Bleeding: above average, manageable without additional treatment than routine coagulation or bone wax Dural closure: unexpected, additional usage of artificial or biological adjunct Repositioning of clip, cage, screw, or rod Technical failure of surgical device MEP: Temporary reduction of amplitude
Grade II	Any deviation from the ideal intraoperative course: • With the need for any additional minor treatment or intervention • Patient with moderate symptoms, not life threatening, and not leading to permanent disability	Bleeding: necessity of clipping, ligature, or local antifibrinolytic agent Accidental opening of sinuses or other cavities needing reconstruction Unplanned extension of craniotomy MEP: reduction of amplitude until end of surgical procedure
Grade III	Any deviation from the ideal intraoperative course: • With the need for any additional moderate treatment or intervention • Patient with severe symptoms, potentially life threatening or potentially leading to permanent disability	Bleeding: with hemodynamic relevance needing immediate surgical and anesthesiologic management Cerebrovascular: rupture of aneurysm or malformation Surgical-induced parenchymal injury MEP: reduction of amplitude up to 50%
Grade IV	Any deviation from the ideal intraoperative course: • With the need for any additional major and urgent treatment or intervention • Patient with life threatening symptoms or leading to permanent disability	Bleeding: necessity of massive transfusion Parenchymal injury of eloquent area or nerval structure leading to permanent disability Intraoperative cerebral edema leading to immediate surgical or anesthesiologic management for ICP reduction MEP: reduction of amplitude over 50%
Grade V	Any deviation from the ideal intraoperative course with intraoperative death of the patient	-

### Primary and secondary outcomes

The primary outcome of this study was the efficacy of the ClassIntra classification to predict postoperative complications. The severity of postoperative complications was graded according to the Clavien-Dindo classification [13], and the Comprehensive Complication Index (CCI) [34]. All complications were listed daily and graded in consensus between three local investigators (R.D, F.L.R, and L.D.). The CCI was calculated as the sum of all complications weighted for their Clavien-Dindo grade according to the publishers’ instructions [34]. We also sought to determine the impact of intraoperative complications on the neurological status of the patients as a secondary outcome. Therefore, patients were evaluated using the Glasgow Coma Scale (GCS) [39], modified Rankin scale (mRS) [28], Neurologic Assessment in Neuro-Oncology (NANO) scale [27], National Institute Health of Strokes Scale (NIHSS) [20], and Karnofsky Performance Score (KPS). These classifications were assessed one day prior surgery and at time of discharge to reflect the possible influence of the intraoperative course. In addition, the need for an unscheduled CT or MRI scan was recorded. Length of hospital and intensive care unit stay was counted from day of surgery until day of discharge from index hospital. Cerebral metastases were divided into groups according to the Recursive Partitioning Analysis (RPA) as published by Radiation Therapy Oncology Group [15], and the Graded Prognostic Assessment (GPA) was done as reported by Sperduto et al. [35] Diagnosis of brain tumors was based on the current 2021 WHO classification for central nervous tumors [25]. Extracranial procedures were defined as any surgery performed without trepanation of the skull bone, such as spinal surgery, and peripheral nerve surgery.

### Statistical analysis

Differences in continuous variables were analyzed with the Mann–Whitney U test and differences in proportions were analyzed with the chi-square-test or Fisher exact test. Univariate and multivariate logistic regression analyses were used to assess the effects of variables and to compute adjusted odds ratio (OR). A two-sided *P* value less than 0.05 was considered as statistically significant. All analyses were performed using SPSS Inc. (V27, Chicago, IL, USA). Data illustrations were performed using GraphPad Prism 9 and Adobe Illustrator 2020.

## Results

**Fig. 1:**
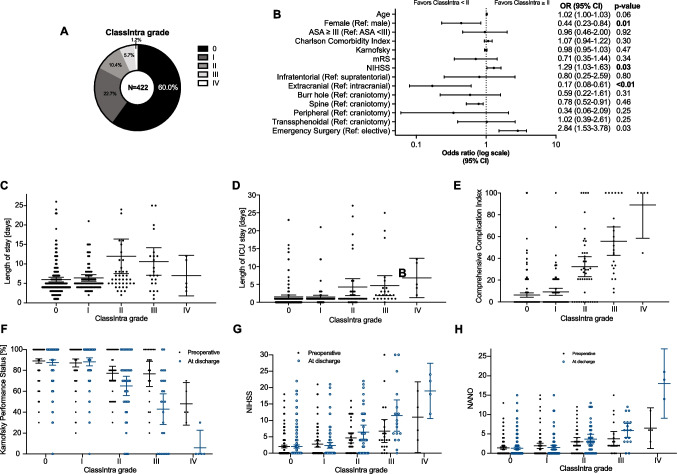
Overview of ClassIntra grade, risk factors, and outcome in all 422 patients who underwent a neurosurgical procedure. **A** Distribution of ClassIntra. **B** Forest plot visualizing covariates with a potential impact on a ClassIntra of II or higher. **C** Length of hospital, **D** ICU stay, and **E** Comprehensive Complication Index for each ClassIntra grade. The neurological outcome was compared between admission to hospital and discharge for each ClassIntra grade and assessed by using **(F**) Karnofsky Performance Status, (**G**) NIHSS, and (**H**) NANO scale

A total of 422 patients were included in this study, of whom 235 (55.7%) were women and 187 (44.3%) were men with a mean age of 56.1 years (Table 2). Patients were stratified according to their ClassIntra grade (Table 2). Of these patients, 324 (76.8%) had a supratentorial pathology and 242 (57.3%) underwent a craniotomy (Table 2). The most procedures were elective (86.5%), while 57 (13.5%) cases were an emergency surgery (Table 2). Among the 422 patients, a deviation from the ideal intraoperative course was noted in 169 (40.0%) cases ranging from severity grade I (22.7%) to IV (1.2%) (Fig. 1A). Patients who had a higher Charlson Comorbidity Index, ASA score, and lower Karnofsky Performance Score were more likely to experience a more severe intraoperative adverse event (Table 2). Furthermore, a poorer neurological status, represented as GCS, mRS, NIHSS, and NANO, showed an increased risk of a higher ClassIntra grade (Table 2). Multivariate analysis was applied to identify risk factors for a ClassIntra grade II or higher (Supplementary table 1). The NIHSS score at admission (OR, 1.29; 95% CI, 1.03–1.63, female gender (OR, 0.44; 95% CI, 0.23–0.84), extracranial procedures (OR, 0.17; 95% CI, 0.08–0.61), and emergency cases (OR, 2.84; 95% CI, 1.53–3.78) were identified as independent factors (Fig. 1B). Among 73 patients with ClassIntra grade II or higher, the length of hospital (*P* < 0.01, Fig. 1C) and ICU (*P* < 0.01, Fig. 1D) stay was significantly longer. The severity of intraoperative adverse events was strongly correlated with postoperative complications and neurological outcome (Fig. 1E-H, Supplementary table 2). This corresponded to a mean (SD) Comprehensive Complication Index of 6.4 (15.9) in patients with no intraoperative adverse events, 9.2 (15.7) in grade I, and raises to 88.9 (24.7) in grade IV (Fig. 1E).

**Table 2:** Clinical features stratified to the ClassIntra grade of all patients who underwent a neurosurgical procedure

Feature	All	Grade 0	Grade I	Grade II	Grade III	Grade IV	*P* value
No., *n* (%)	422	253 (60.0)	96 (22.7)	44 (10.4)	24 (5.7)	5 (1.2)	
Age [years], mean (SD)	56.1 (16.2)	55.7 (16.1)	54.5 (16.8)	60.9 (16.8)	55.5 (14.1)	64.8 (23.3)	.17
Gender, *n* (%)							
Female	235 (55.7)	150 (63.8)	55 (23.4)	18 (7.7)	10 (4.3)	2 (0.9)	.10
Male	187 (44.3)	103 (55.1)	41 (21.9)	26 (13.9)	14 (7.5)	3 (1.6)	
ASA, *n* (%)							
I	17 (4.0)	11 (64.7)	5 (29.4)	1 (5.9)	0 (0.0)	0 (0.0)	** < .01**
II	209 (49.5)	130 (62.2)	50 (23.9)	18 (8.6)	11 (5.3)	0 (0.0)	
III	151 (35.8)	92 (60.9)	36 (23.8)	17 (11.3)	5 (3.3)	1 (0.7)	
IV	21 (5.0)	10 (47.6)	4 (19.0)	2 (9.5)	3 (14.3)	2 (9.5)	
V	24 (5.7)	10 (41.7)	1 (4.2)	6 (25.0)	5 (20.8)	2 (8.3)	
BMI, mean (SD)	26.7 (5.5)	26.4 (5.6)	27.1 (4.8)	27.4 (4.8)	27.0 (6.7)	26.3 (1.9)	.82
Charlson Comorbidity index, mean (SD)	3.3 (2.9)	3.2 (2.7)	2.8 (2.6)	3.8 (2.9)	4.7 (3.8)	5.4 (5.5)	**.01**
Karnofsky, mean (SD)	86.5 (18.8)	89.0 (16.8)	87.1 (18.8)	77.3 (20.6)	76.7 (26.5)	50.0 (14.1)	** < .01**
Glasgow coma scale, mean (SD)	14.6 (1.4)	14.8 (0.8)	14.7 (1.2)	14.1 (2.2)	13.6 (2.9)	9.5 (4.9)	** < .01**
Modified Rankin scale, mean (SD)	0.9 (1.2)	0.7 (1.1)	0.8 (1.2)	1.3 (1.5)	1.6 (1.6)	3.5 (0.7)	** < .01**
NIHSS, mean (SD)	2.8 (4.3)	2.0 (3.4)	2.8 (4.6)	4.7 (4.8)	6.8 (7.7)	10.5 (4.8)	** < .01**
NANO, mean (SD)	1.8 (2.8)	1.4 (2.2)	1.9 (3.3)	2.9 (3.3)	3.8 (4.1)	8.5 (3.5)	** < .01**
Urgency, *n* (%)							
Elective	365 (86.5)	222 (60.8)	91 (24.9)	34 (9.3)	18 (5.0)	0 (0.0)	** < .01**
Emergency	57 (13.5)	31 (54.4)	5 (8.8)	10 (17.5)	6 (10.5)	5 (8.8)	
Location, *n* (%)							
Supratentorial	324 (76.8)	188 (58.0)	73 (22.5)	38 (11.7)	20 (6.2)	5 (1.5)	** < .01**
Infratentorial	35 (8.3)	13 (37.1)	17 (48.6)	4 (11.4)	1 (2.9)	0 (0.0)	
Spine	27 (6.4)	18 (66.7)	5 (18.5)	1 (3.7)	3 (11.1)	0 (0.0)	
Peripheral	36 (8.5)	34 (94.4)	1 (2.8)	1 (2.8)	0 (0.0)	0 (0.0)	
Approach, *n* (%)							
Craniotomy	242 (57.3)	120 (49.6)	72 (29.8)	30 (12.4)	15 (6.2)	5 (2.1)	** < .01**
Burr hole	53 (12.6)	36 (67.9)	7 (13.2)	6 (11.3)	4 (7.5)	0 (0.0)	
Transsphenoidal	55 (13.0)	40 (72.7)	4 (8.7)	2 (4.3)	2 (3.6)	0 (0.0)	
Spine	26 (6.2)	17 (65.4)	5 (19.2)	1 (3.8)	3 (11.5)	0 (0.0)	
Peripheral	46 (10.9)	40 (87.0)	4 (8.7)	2 (4.3)	0 (0.0)	0 (0.0)	
Position, *n* (%)							
Supine	326 (77.3)	200 (61.3)	68 (20.9)	34 (10.4)	19 (5.8)	5 (1.5)	.11
Semi-lateral/lateral	54 (12.8)	29 (53.7)	15 (27.8)	9 (16.7)	1 (1.8)	0 (0.0)	
Prone	41 (9.7)	24 (58.5)	12 (29.3)	1 (2.4)	4 (9.8)	0 (0.0)	
Sitting	1 (0.2)	0 (0.0)	1 (100.0)	0 (0.0)	0 (0.0)	0 (0.0)	
Indication/Procedure, *n* (%)							
Abscess formation	5 (1.2)	3 (60.0)	0 (0.0)	0 (0.0)	1 (20.0)	1 (20.0)	** < .01**
Intracranial haemorrhage	28 (6.6)	17 (60.8)	2 (7.1)	5 (17.9)	2 (7.1)	2 (7.1)	
Biopsy of unclear lesion	18 (4.3)	12 (66.7)	3 (16.7)	2 (11.1)	1 (5.6)	0 (0.0)	
Disc herniation/spinal stenosis	10 (2.4)	5 (50.0)	4 (40.0)	0 (0.0)	1 (10.0)	0 (0.0)	
Cavernoma	4 (0.9)	1 (25.0)	3 (75.0)	0 (0.0)	0 (0.0)	0 (0.0)	
Cerebrovascular	44 (10.4)	22 (50.0)	15 (34.1)	5 (11.4)	1 (2.3)	1 (2.3)	
Brain tumour	125 (29.6)	64 (51.2)	38 (30.4)	16 (12.8)	7 (5.6)	0 (0.0)	
Hemangioblastoma	3 (0.7)	1 (33.3)	2 (66.7)	0 (0.0)	0 (0.0)	0 (0.0)	
Hemicraniectomy	7 (1.7)	3 (42.9)	1 (14.3)	1 (14.3)	2 (28.6)	0 (0.0)	
Intraspinal tumour	15 (3.6)	11 (73.3)	1 (6.7)	1 (6.7)	2 (13.3)	0 (0.0)	
Suprasellar lesion	57 (13.5)	40 (70.2)	9 (15.8)	5 (8.8)	3 (5.3)	0 (0.0)	
Vestibular schwannoma	9 (2.1)	1 (11.1)	4 (44.4)	3 (33.3)	1 (11.1)	0 (0.0)	
Hydrocephalus	41 (9.7)	27 (65.9)	5 (12.2)	5 (12.2)	3 (7.3)	1 (2.4)	
Peripheral nerve tumour	27 (6.4)	27 (100.0)	0 (0.0)	0 (0.0)	0 (0.0)	0 (0.0)	
Bone flap replacement	4 (0.9)	1 (25.0)	3 (75.0)	0 (0.0)	0 (0.0)	(0.0)	
Temporal lobe epilepsy	10 (2.4)	6 (60.0)	4 (40.0)	0 (0.0)	0 (0.0)	0 (0.0)	
Trigeminal neuralgia	8 (1.9)	8 (100.0)	0 (0.0)	0 (0.0)	0 (0.0)	0 (0.0)	
Others	7 (1.7)	4 (57.1)	2 (28.6)	1 (14.3)	0 (0.0)	0 (0.0)	
Operating duration [min], mean (SD)	127.5 (83.9)	104.5 (70.7)	165.7 (80.0)	151.1 (101.9)	173.0 (113.2)	116.0 (195.6)	** < .01**

*P* values reaching a significance level below 0.05 are marked bold

### Elective craniotomies

**Fig. 2:**
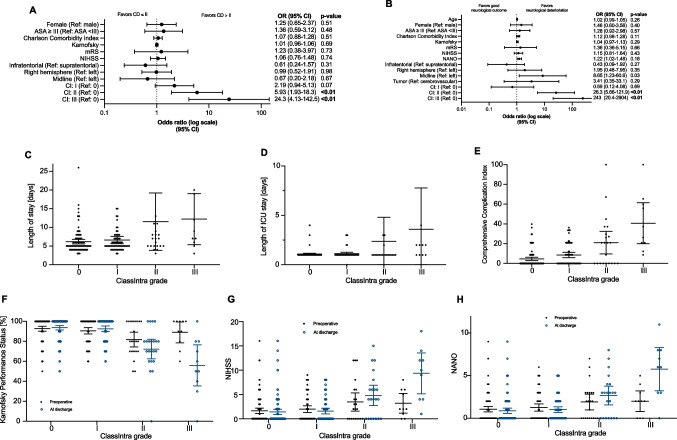
Visualization of postoperative outcome in 209 patients who underwent elective craniotomy. Forest plots representing covariates for (**A**) a higher Clavien-Dindo ≥ II, and (**B**) neurological deterioration at discharge. (**C**) Length of hospital, (**D**) ICU stay, and (**E**) Comprehensive Complication Index for each ClassIntra grade. The neurological outcome was compared between admission to hospital and discharge for each ClassIntra grade and assessed by using (**F**) Karnofsky Performance Status, (**G**) NIHSS, and (**H**) NANO scale

For the 209 patients who underwent an elective craniotomy, a deviation from the ideal intraoperative course was registered in 101 (48.3%) patients (Table 3). Most of the cases were supratentorial (84.2%) with brain tumors (63.2%) and cerebrovascular disorders (17.2%) being the most common pathologies (Table 3). A higher Charlson Comorbidity Index, and lower Karnofsky Performance Score at admission significantly increased the risk for an intraoperative adverse event (Table 3). Focusing on the outcome, a higher ClassIntra grade resulted in more severe postoperative complications as reflected by the Clavien-Dindo classification (*P* < 0.01, Fig. 2A) and Comprehensive Complication Index (*P* < 0.01, Fig. 2E). Among these patients experiencing an intraoperative adverse event, the probability for a deterioration of the neurological status between admission and discharge was significantly increased (Fig. 2F-H). A ClassIntra grade of II or higher could be verified as an independent factor for an unfavorable neurological outcome (OR, 26.3; 95% CI, 5.66–121.9, Fig. 2B, Supplementary table 3) and postoperative complications of CD > II (OR, 5.93; 95% CI, 1.93–18.3, Fig. 2A, Supplementary table 4).

**Table 3:** Clinical features and outcome stratified to the ClassIntra grade of patients who underwent an elective craniotomy

Feature	All	Grade 0	Grade I	Grade II	Grade III	*P* value
No., *n* (%)	209	108 (51.7)	69 (33.0)	22 (10.5)	10 (4.8)	
Age [years], mean (SD)	55.2 (15.9)	54.4 (15.8)	54.2 (16.6)	63.1 (13.9)	52.6 (13.7)	.11
Gender, *n* (%)						
Female	126 (60.3)	69 (54.8)	41 (32.5)	11 (8.7)	5 (4.0)	.57
Male	83 (39.7)	39 (47.0)	28 (33.7)	11 (13.3)	5 (6.0)	
ASA, *n* (%)						
I	7 (3.3)	4 (57.1)	3 (42.9)	0 (0.0)	0 (0.0)	.19
II	121 (57.9)	68 (56.2)	37 (30.6)	10 (8.3)	6 (5.0)	
III	78 (37.3)	36 (46.2)	28 (35.9)	11 (14.1)	3 (3.8)	
IV	3 (1.4)	0 (0.0)	1 (33.3)	1 (33.3)	1 (33.3)	
BMI, mean (SD)	26.9 (5.7)	26.5 (5.9)	27.2 (5.6)	27.6 (5.1)	22.7 (2.9)	.14
Anticoagulation, *n* (%)						
None	180 (86.1)	99 (55.0)	56 (31.1)	17 (9.4)	8 (4.4)	.08
Paused	18 (8.6)	7 (38.9)	8 (44.4)	3 (16.7)	0 (0.0)	
Under medication	11 (5.3)	2 (18.2)	5 (45.5)	2 (18.2)	2 (18.2)	
Charlson Comorbidity index, mean (SD)	3.3 (2.9)	3.2 (2.8)	2.8 (2.6)	4.3 (3.3)	5.0 (3.8)	**.04**
Karnofsky, mean (SD)	90.7 (13.6)	92.7 (12.6)	90.6 (13.1)	81.8 (16.8)	89.0 (14.5)	** < .01**
Glasgow coma scale, mean (SD)	14.9 (0.4)	14.9 (0.3)	14.9 (0.12)	14.8 (0.8)	14.8 (0.6)	.08
Modified Rankin scale, mean (SD)	0.6 (0.9)	0.5 (0.9)	0.6 (0.8)	1.1 (1.2)	0.9 (1.1)	.07
NIHSS, mean (SD)	2.04 (3.1)	1.67 (2.9)	2.0 (2.8)	3.45 (4.2)	3.20 (2.8)	.05
NANO, mean (SD)	1.3 (1.8)	1.1 (1.6)	1.3 (1.7)	1.9 (2.1)	2.0 (1.7)	.10
Hemoglobin, mean (SD)	13.8 (1.7)	13.6 (1.6)	14.2 (1.6)	13.9 (1.6)	12.2 (2.6)	**< .01**
Platelet, mean (SD)	263 (79)	266 (81)	262 (80)	257 (72)	259 (67)	.95
Location, *n* (%)						
Supratentorial	176 (84.2)	96 (54.5)	52 (29.5)	19 (10.8)	9 (5.1)	.25
Infratentorial	33 (15.8)	12 (36.3)	17 (51.5)	3 (9.1)	1 (3.1)	
Side, *n* (%)						
Left	88 (42.1)	46 (52.3)	26 (29.5)	12 (13.6)	4 (4.5)	.56
Right	100 (47.8)	54 (54.0)	35 (35.0)	7 (7.0)	4 (4.0)	
Midline	21 (10.0)	8 (38.1)	8 (38.1)	3 (14.3)	2 (9.5)	
Approach, *n* (%)						
Frontal	37 (17.7)	19 (51.4)	9 (24.3)	5 (13.5)	4 (10.8)	.78
Pterional/temporal	82 (39.2)	43 (52.4)	30 (36.6)	7 (8.5)	2 (2.4)	
Suboccipital	29 (13.9)	12 (41.4)	12 (41.4)	3 (10.3)	2 (6.9)	
Retrosigmoidal	25 (12.0)	13 (52.0)	8 (32.0)	3 (12.0)	1 (4.0)	
Parietooccipital	36 (17.2)	21 (58.3)	10 (27.8)	4 (11.1)	1 (2.8)	
Position, *n* (%)						
Supine	145 (69.4)	77 (53.1)	46 (31.7)	14 (9.7)	8 (5.5)	.16
Semi-lateral/lateral	49 (23.4)	25 (51.0)	15 (30.6)	8 (16.3)	1 (2.1)	
Prone	14 (6.7)	6 (42.9)	7 (50.0)	0 (0.0)	1 (7.1)	
Sitting	1 (0.5)	0 (0.0)	1 (100.0)	0 (0.0)	0 (0.0)	
Electrophysiological monitoring, *n* (%)						
No	179 (85.6)	101 (56.4)	53 (29.6)	18 (10.1)	7 (3.9)	** < .01**
Yes	30 (14.4)	7 (23.3)	16 (53.3)	4 (13.3)	3 (10.0)	
Indication, *n* (%)						
Abscess formation	2 (1.0)	1 (50.0)	0 (0.0)	0 (0.0)	1 (50.0)	**.02**
Selective Amygdalohippocampectomy	9 (4.3)	5 (55.6)	4 (44.4)	0 (0.0)	0 (0.0)	
Biopsy of unclear lesion	5 (2.4)	3 (60.0)	1 (20.0)	1 (20.0)	0 (0.0)	
Cerebrovascular	36 (17.2)	19 (52.8)	14 (38.9)	3 (8.3)	0 (0.0)	
Brain tumour	123 (58.9)	64 (52.0)	37 (30.1)	15 (12.2)	7 (5.7)	
Vestibular schwannoma	9 (4.3)	1 (11.1)	4 (44.4)	3 (33.3)	1 (11.1)	
Trigeminal neuralgia	7 (3.3)	7 (100.0)	0 (0.0)	0 (0.0)	0 (0.0)	
Other	18 (8.6)	8 (44.4)	9 (50.0)	0 (0.0)	1 (5.6)	
Operating duration [min], mean (SD)	177 (80)	157 (65)	189 (76)	212 (98)	233 (139)	** < .01**
Outcome						
Unscheduled CT/MRI scan, *n* (%)	35 (16.7)	10 (9.3)	10 (14.5)	8 (36.4)	7 (70.0)	** < .01**
Reoperation, *n* (%)	9 (4.3)	3 (2.8)	3 (4.3)	2 (9.1)	1 (10.0)	.45
Highest Clavien-Dindo grade, *n* (%)						
0	125 (59.8)	79 (73.1)	37 (53.6)	9 (40.9)	0 (0.0)	** < .01**
I	31 (14.8)	14 (13.0)	14 (20.3)	1 (4.5)	2 (20.0)	
II	39 (18.7)	14 (13.0)	14 (20.3)	8 (36.4)	3 (30.0)	
IIIa	3 (1.4)	0 (0.0)	1 (1.4)	1 (4.5)	1 (10.0)	
IIIb	7 (3.3)	1 (0.9)	3 (4.3)	1 (4.5)	2 (20.0)	
IVa	2 (1.0)	0 (0.0)	0 (0.0)	1 (4.5)	1 (10.0)	
IVb	0 (0.0)	0 (0.0)	0 (0.0)	0 (0.0)	0 (0.0)	
V	2 (1.0)	0 (0.0)	0 (0.0)	1 (4.5)	1 (10.0)	
Comprehensive Complication Index, mean (SD)	9.3 (16.1)	4.6 (9.0)	8.5 (11.1)	20.9 (25.6)	40.7 (28.9)	** < .01**
Karnofsky worsening, *n* (%)	27 (12.9)	6 (5.6)	2 (2.9)	10 (45.5)	9 (90.0)	** < .01**
GCS worsening, *n* (%)	9 (4.3)	1 (0.9)	0 (0.0)	2 (9.1)	6 (60.0)	** < .01**
mRS worsening, *n* (%)	27 (12.9)	5 (4.6)	3 (4.3)	10 (45.5)	9 (90.0)	** < .01**
NIHSS worsening, *n* (%)	29 (13.9)	5 (4.6)	4 (5.8)	11 (50.0)	9 (90.0)	** < .01**
NANO worsening, *n* (%)	32 (15.3)	7 (6.5)	6 (8.7)	10 (45.5)	9 (90.0)	** < .01**
Length of ICU stay, mean (SD)	1.4 (2.3)	1.1 (0.5)	1.1 (0.5)	2.4 (5.5)	3.6 (5.8)	** < .01**
Length of hospital stay, mean (SD)	7.2 (7.0)	6.2 (3.2)	6.6 (4.1)	11.6 (17.4)	12.2 (9.6)	** < .01**

*P* values reaching a significance level below 0.05 are marked bold

### Brain tumor surgery

**Fig. 3:**
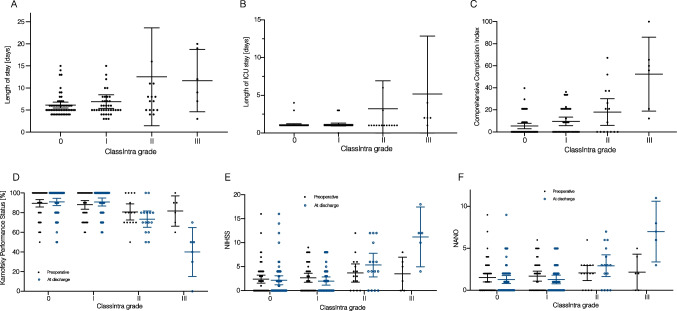
Overview of ClassIntra grade and outcome in 118 patients who underwent elective brain tumor surgery. **A** Length of hospital, **B** ICU stay, and **C** Comprehensive Complication Index for each ClassIntra grade. The neurological outcome was compared between admission to hospital and discharge for each ClassIntra grade and assessed by using (**D**) Karnofsky Performance Status, (**E**) NIHSS, and (**F**) NANO scale

Of the 209 patients who underwent an elective craniotomy, we performed further analyses on 118 (56.4%) patients with an intra- or extra-axial brain tumor (Supplementary table 5). An intraoperative adverse event was detected in 61 (51.7%) surgeries. Type and diameter of the tumor were not predictive for an intraoperative adverse event, but eloquent location (*p* = 0.03) was associated with a higher ClassIntra (Supplementary table 5). Patients with a ClassIntra ≥ II had a significantly longer length of hospital stay (*P* < 0.01, Fig. 3C), ICU stay (*P* < 0.01, Fig. 3D), and suffered from higher morbidity and mortality (*P* < 0.01, Fig. 3E). In addition, these patients were more favorable for a neurological deterioration (Fig. 3F-H). These results could be verified for the brain tumor subgroups including metastases, meningioma, and glioma (Supplementary tables 6, 7 and 8).

For cerebral metastases, the type of primary tumor (*P* = 0.27), number of resected metastases (*P* = 0.42), RPA (*p* = 0.34), and GPA score (*P* = 0.23) did not influence the severity of intraoperative adverse events (Supplementary table 6). Furthermore, there were no meningioma- (e.g., Simpson grade, WHO grade, sinus infiltration) and glioma-specific (histology, WHO grade, extent of resection) characteristics predictive for ClassIntra (Supplementary tables 7 and 8).

### Cerebrovascular surgery

We identified 45 patients who underwent craniotomy for microsurgical clipping of an intracranial aneurysm. Of these, 7 (15.6%) patients suffered from subarachnoid hemorrhage due to a ruptured aneurysm. Here, we focused on 38 (84.4%) patients with an unruptured intracranial aneurysm (UIA) and analyzed for aneurysm-specific risk factors for intraoperative adverse events (Supplementary table 9). Overall, 16 (51.6%) patients had no intraoperative adverse event, while 12 (38.7%) had a ClassIntra I and 3 (9.7%) patients had a ClassIntra II. Aneurysm location, PHASES score, largest diameter, and calcification did not increase the risk of intraoperative adverse events (Supplementary table 9). However, the ClassIntra grade correlated with the aneurysm morphology (*P* = 0.01) and number of clips needed for aneurysm occlusion (*P* = 0.02). Patients with a higher ClassIntra had a more severe postoperative complication as shown by Clavien-Dindo (*P* = 0.04) and Comprehensive Complication Index (*P* = 0.02). Contrary to the previous results, the length of ICU and hospital stay did not differ between these groups (Supplementary table 9). An aneurysm remnant in early postsurgical CT angiography did not correlate with intraoperative adverse events (*P* = 0.09).

### Emergency craniotomies

A subgroup of 32 patients who underwent urgent craniotomy due to an emergency was created to analyze the reliability of the ClassIntra in this specific situation (Supplementary table 10). Of these 32 patients, the main procedure was hematoma evacuation (46.9%) and microsurgical clipping of a ruptured aneurysm (21.9%). Preoperative neurological status, and ventilated situation at hospital admission did not influence the severity of the intraoperative adverse events (Supplementary table 10). The proportion of a severe intraoperative adverse event classified as ClassIntra grade II, III, or IV was significantly higher than in the elective craniotomy group (56.2% versus 15.3%). A higher ClassIntra correlated with more severe complications, in-hospital mortality, and an unfavorable outcome at discharge (Supplementary table 10).

## Discussion

The importance of a standardized report system for intraoperative adverse events has been emphasized repeatedly [12, 18] ^(p)^. Despite an increasing effort in the past years, no standardized grading system for assessing intraoperative complications has been implemented into the daily routine in the operating room. The introduction and validation of the Classification of Intraoperative Adverse Events (ClassIntra) is one promising approach to close this gap but its potential in the neurosurgical field is unknown [11]. In this context, our study presents the following major findings: 1) A deviation from the ideal intraoperative course was noted in 40.0% of neurosurgical procedures with an increased risk for an event in intracranial pathologies of comorbid patients. 2) For elective craniotomies, a ClassIntra grade of II or higher is strongly associated with more severe postoperative complications, a neurological deterioration at discharge, and prolonged ICU and hospital stay. These results were also valid for patient subgroups who underwent elective brain tumor and cerebrovascular surgery, but disease-related characteristics for predicting the severity of intraoperative adverse events could not be identified. 3) Emergency craniotomies were at higher risk for intraoperative adverse events, and the severity of ClassIntra correlated with postoperative complications and in-hospital mortality*.*

For improving patient’s outcome and reducing morbidity as well as mortality after surgical procedures in the future, a standardized system to measure current complications is indispensable. Even though there are accepted classification systems for scoring comorbidities [7], and postoperative complications [13, 34], the intraoperative course could not be well-reflected in a standardized manner by now. Dell-Kuster and colleagues presented the ClassIntra and proved its reliability to assess intraoperative adverse events and predict postoperative complications across different surgical disciplines [11] ^(p)^. Further studies could show a high inter-rater agreement and correlation with postoperative complications in elective abdominal [22] and ophthalmological surgery [5]. Nevertheless, the potential role of the ClassIntra for neurosurgical procedures remains elusive. It must be mentioned that the validation cohort by Dell-Kuster et al. included 96 neurosurgical and spine procedures, but more detailed information on these patients were not given [11] ^(p)^. Our prospective study covered a broad spectrum of neurosurgical procedures with a main focus on craniotomies (57.3%) as these are constituted as the most complex in the neurosurgical field. In general, a deviation from the ideal intraoperative course was noted in 40.0% of the procedures with a higher risk for events in elective (48.3%) and emergent craniotomies (56.2%). These results are comparable with those of the neurosurgical and spine patients (44.8% intraoperative adverse events) described by Dell-Kuster et al [11] ^(p)^. It must be mentioned that most of the documented intraoperative adverse events (56.8%) were graded as I without needing an additional intervention during surgery and having an identical outcome than patients graded as 0. In our study, in 17.3% of the procedures occurred an intraoperative adverse event which was followed by a minor or moderate treatment intraoperative. As the frequency of the intraoperative adverse events are comparable between ours and Dell-Kusters cohort, the ClassIntra seems to be transferable and reliable across various departments of the same surgical discipline. The clearly defined grading criteria are a major contributor for the easily possible integration of ClassIntra into clinical routine, and has a huge potential to establish its role as a widely accepted classification for assessing intraoperative adverse events, as current reports did not use standardized grading systems or were limited to specific procedures [29, 43].

A major finding of the study by Dell-Kuster and colleagues was the increase in risk for a more severe postoperative complication and length of hospital stay with an increasing grade of ClassIntra [11]. In our study, we experienced a close relation between the ClassIntra grade and the postoperative outcome as well. Neurosurgical patients experiencing an intraoperative adverse event graded as II or higher were more likely to have more severe complications, a higher comprehensive complication index, and longer ICU and hospital stay. In addition, this correlated with the need of unscheduled CT or MRI scans. To underscore the value, we could verify these findings on several patient subgroups including elective and emergent craniotomies as well as cerebrovascular and brain tumor surgery. As already mentioned, the neurological outcome of neurosurgical patients is from particular importance. Bearing this in mind, our study revealed a strong correlation between a more severe ClassIntra and a deterioration of various neurological scores between admission and discharge emphasizing the value of ClassIntra. For elective craniotomies, ClassIntra could be identified as an independent factor for predicting an unfavorable neurological outcome.

To date, no standardized grading system for assessing intraoperative complications is integrated into the daily clinical routine. However, Gozal and colleagues introduced a promising complication classification which reflects pre-, peri- and postoperative adverse events and categorizes into five types of errors [17]. The proposed classification system has its strength in detecting errors as a source of the complication and forms the basis to avoid these errors in the future [17]. Nevertheless, the ClassIntra takes surgical as well as anesthesiologic complications into perspective. While the ClassIntra assesses intraoperative adverse events to predict the postoperative risk of potential complications, the complication classification by Gozal et al. offers a framework for teaching, and institutional quality improvement. Although ClassIntra classification offers many advantages in the assessment of neurosurgical and anesthesiologic adverse events, postoperative deficits may result from factors other than surgical problems, such as lesioning of the corticospinal tract. Furthermore, an intraoperative decision may be made in favor of an increased extent of resection with, however, a worse neurological outcome in the neuro-oncological field, the assignment of which to the ClassIntra classification remains to be discussed in the future. It would be conceivable to add a subcategory within the ClassIntra classification for these special cases or to combine the classification with an existing postoperative score, such as the TDN classification [40].

In the past years, the need for ICU admission after elective craniotomies attached greater importance and several studies aimed to define risk profiles for postoperative complications [1]. In this context, protocols and trials for enhanced recovery after surgery have found their way into the neurosurgical field even though craniotomies are still considered as high risk procedures [14, 36, 41]. For stratifying patients according to their risk profile, intraoperative criteria included type of lesion, length of surgery, excessive bleeding, and diabetes insipidus [1]. In addition, various preoperative risk factors such as diabetes mellitus for epilepsy surgery were defined [2]. It is well known that postsurgical monitoring in an ICU causes higher costs, therefore, finding the balance between patients safety and cost-effectiveness is particularly important [3, 24]. The integration of ClassIntra seems to offer an ideal possibility for identifying patients with high risk for postoperative complications and deciding the type of postoperative care. Nevertheless, further predictors of deviation from the ideal intraoperative course need to be found in future studies and preoperative risk stratification must be optimized.

Undoubtably, this study has some limitations. The evaluation of the ClassIntra was made according to the previously published categorization but could be influenced by the surgeons and anesthesiologists’ perspective on the severity of the intraoperative complication. In addition, we did not take the surgeons and anesthesiologists experience into perspective on the grounds of data protection which could be an influencing factor. Additionally, the cohorts for the subgroup entities are comparatively small, which should be considered when interpreting the statistical analyses. Lastly, all data are gathered from a monocentric cohort and are not validated on an external patient’s cohort.

## Conclusion

In this monocentric, prospective study assessing intraoperative adverse events by using the ClassIntra in a wide spectrum of neurosurgical procedures, the classification was highly predictive for postoperative complications, length of hospital stay, and neurological deterioration at discharge. These findings were reproducible for elective and emergency craniotomy as well as brain tumor and cerebrovascular surgery. Overall, the ClassIntra is a simply applicable classification for recording intraoperative adverse events and identifying patients with a high risk for postoperative complications depending on the intraoperative course.

## Supplementary Information

**Additional file 1. (PDF 404 KB)**

## Data Availability

Data are available upon reasonable request.
