# Supplementary material for: Association of the classification of intraoperative adverse events (ClassIntra) with complications and neurological outcome after neurosurgical procedures: a prospective cohort study
Source: Acta Neurochir (Wien). 2023 Jul 5;165(8):2015–27. doi: 10.1007/s00701-023-05672-w (PMC10409660; doi:10.1007/s00701-023-05672-w)
Supplement: Supplementary file 1 — Additional file 1. (PDF 404 KB) [file 701_2023_5672_MOESM1_ESM.pdf]

Supplementary table 1: Uni- and multivariate analysis identifying risk factors for a ClassIntra grade of II or higher.

Feature	Univariate		Multivariate	
	OR (95%-CI)	p-value	OR (95%-CI)	*P* value
**Age**	1.02 (1.00-1.03)	.06		
**Gender**				
Male	*Ref.*		*Ref.*	
Female	0.49 (0.29-0.81)	**<.01**	0.44 (0.23-0.84)	**.01**
**ASA**				
≤II	Ref.		Ref.	
≥III	1.83 (1.09-3.05)	**.02**	0.96 (0.46-2.00)	.92
**BMI**	1.02 (0.97-1.07)	.47		
**Anticoagulation**				
None	*Ref.*		*Ref.*	
Paused	0.69 (0.23-2.02)	.49	0.35 (0.09-1.33)	.12
Under medication	2.23 (1.09-4.54)	**.03**	0.89 (0.30-2.61)	.82
**Charlson Comorbidity Index**	1.14 (1.05-1.24)	**<.01**	1.07 (0.94-1.22)	.30
**Karnofsky**	0.97 (0.96-0.98)	**<.01**	0.98 (0.95-1.03)	.47
**Glasgow coma scale**	0.69 (0.59-0.82)	**<.01**	0.78 (0.54-1.13)	.19
**Modified Rankin scale**	1.52 (1.25-1.85)	**<.01**	0.71 (0.35-1.44)	.34
**NIHSS**	1.15 (1.09-1.21)	**<.01**	1.29 (1.03-1.63)	**.03**
**NANO**	1.19 (1.10-1.29)	**<.01**	0.81 (0.55-1.21)	.30
**Location**				
Supratentorial	*Ref.*		*Ref.*	
Infratentorial	0.71 (0.26-1.90)	.49	0.80 (0.25-2.59)	.80
Spine / extracranial	0.29 (0.10-0.84)	**.02**	0.17 (0.08-0.61)	**<.01**
**Approach**				
Craniotomy	*Ref.*		*Ref.*	
Burr hole	0.89 (0.41-1.89)	.76	0.59 (0.22-1.61)	.31
Spine	0.69 (0.23-2.11)	.52	0.78 (0.52-0.91)	.46
Peripheral	0.17 (0.04-0.74)	**.02**	0.34 (0.06-2.09)	.25
Transsphenoidal	0.56 (0.24-1.31)	.18	1.02 (0.39-2.61)	.25
**Urgency**				
Elective	*Ref.*		*Ref.*	
Emergency	3.50 (1.89-6.46)	**<.01**	2.84 (1.53-3.78)	**.03**
**Position**				
Supine	*Ref.*			
Semi-lateral/lateral	1.05 (0.50-2.21)	.89		
Prone	0.64 (0.24-1.71)	.37		

Supplementary table 2: Postoperative outcome of all patients who underwent a neurosurgical procedure.

Feature	All	Grade 0	Grade I	Grade II	Grade III	Grade IV	*P* value
No., n (%)	422	253 (60.0)	96 (22.7)	44 (10.4)	24 (5.7)	5 (1.2)	
Unscheduled CT/MRI scan, n (%)	73 (17.3)	20 (7.9)	12 (12.5)	19 (43.2)	18 (75.0)	4 (80.0)	**<.01**
Reoperation, n (%)	28 (6.6)	5 (2.0)	4 (4.2)	11 (25.0)	6 (25.0)	2 (40.0)	**<.01**
Highest Clavien-Dindo grade, n (%)							
0	259 (61.4)	192 (75.9)	55 (57.3)	12 (27.3)	0 (0.0)	0 (0.0)	**<.01**
I	41 (9.7)	20 (7.9)	17 (17.7)	2 (4.5)	2 (8.3)	0 (0.0)	
II	65 (15.4)	29 (11.5)	17 (17.7)	15 (34.1)	4 (16.7)	0 (0.0)	
IIIa	7 (1.7)	2 (0.8)	1 (1.0)	1 (2.3)	3 (12.5)	0 (0.0)	
IIIb	19 (4.5)	2 (0.8)	4 (4.2)	7 (15.9)	5 (20.8)	1 (20.0)	
IVa	11 (2.6)	4 (1.6)	0 (0.0)	3 (6.8)	4 (16.7)	0 (0.0)	
IVb	1 (0.2)	0 (0.0)	1 (1.0)	0 (0.0)	0 (0.0)	0 (0.0)	
V	19 (4.5)	4 (1.6)	1 (1.0)	4 (9.1)	6 (25.0)	4 (80.0)	
Comprehensive Complication Index, mean (SD)	13.5 (24.5)	6.4 (15.9)	9.2 (15.7)	32.3 (30.1)	55.8 (31.1)	88.9 (24.7)	**<.01**
Karnofsky, mean (SD)	86.1 (20.1)	89.5 (17.1)	89.2 (16.7)	71.8 (22.7)	57.2 (27.6)		**<.01**
Karnofsky worsening, n (%)	60 (14.7)	12 (4.9)	4 (4.2)	19 (47.5)	20 (87.0)	5 (100.0)	**<.01**
Glasgow coma scale, mean (SD)	14.6 (1.4)	14.8 (0.8)	14.8 (0.8)	14.2 (1.8)	12.4 (4.1)		**<.01**
Glasgow coma scale worsening, n (%)	34 (8.3)	6 (2.4)	1 (1.0)	9 (22.5)	13 (56.5)	5 (100.0)	**<.01**
Modified Rankin scale, mean (SD)							
Modified Rankin scale worsening, n (%)	57 (13.9)	9 (3.7)	5 (5.2)	18 (45.0)	20 (87.0)	5 (100.0)	**<.01**
NIHSS, mean (SD)	3.0 (5.2)	2.0 (3.9)	2.4 (4.3)	6.4 (6.6)	11.5 (9.0)		**<.01**
NIHSS worsening, n (%)	62 (15.2)	11 (4.5)	7 (7.3)	19 (47.5)	20 (87.0)	5 (100.0)	**<.01**
NANO, mean (SD)	1.8 (2.9)	1.3 (2.4)	1.5 (2.8)	3.7 (3.8)	5.9 (3.6)		**<.01**
NANO worsening, n (%)	63 (15.5)	13 (5.3)	8 (8.3)	18 (45.0)	19 (86.4)	5 (100.0)	**<.01**
Length of ICU stay, mean (SD)	2.0 (4.7)	1.5 (4.3)	1.5 (2.7)	4.3 (7.8)	4.7 (6.6)	6.8 (4.4)	**<.01**
Length of hospital stay, mean (SD)	6.9 (68)	5.9 (4.9)	6.4 (4.2)	11.9 (14.6)	10.6 (8.4)	7 (4.2)	**<.01**

Supplementary table 3: Uni- and multivariate analysis identifying risk factors for an unfavourable outcome at discharge after elective craniotomy. An unfavourable outcome is defined as a simultaneous worsening of Karnofsky, mRS, NIHSS, and NANO scale. The analyses included 209 patients, of whom 25 had an unfavourable outcome.

Feature	Univariate		Multivariate	
	OR (95%-CI)	p-value	OR (95%-CI)	*P* value
**Age**	1.02 (0.99-1.05)	.26		
**Gender**				
Male	Ref.			
Female	1.46 (0.60-3.56)	.40		
**ASA**				
I / II	Ref.			
III / IV	1.28 (0.55-2.98)	.57		
**BMI**	0.99 (0.92-1.08)	.98		
**Anticoagulation**				
None	Ref.			
Paused	0.45 (0.06-3.52)	.44		
Under medication	2.84 (0.69-11.5)	.15		
**Charlson Comorbidity Index**	1.12 (0.98-1.28)	.11		
**Karnofsky**	0.98 (0.95-1.01)	**.03**	1.04 (0.97-1.13)	.29
**Modified Rankin scale**	1.54 (1.03-2.29)	**.03**	1.36 (0.36-5.15)	.66
**NIHSS**	1.15 (1.03-1.29)	**.02**	1.15 (0.81-1.64)	.43
**NANO**	1.27 (1.03-1.55)	**.02**	1.22 (1.02-1.48)	.18
**Location**				
Supratentorial	Ref.			
Infratentorial	0.43 (0.09-1.92)	.27		
**Side**				
Left	Ref.		Ref.	
Right	0.98 (0.38-2.52)	.96	1.95 (0.48-7.95)	.35
Midline	3.51 (1.09-11.3)	**.04**	8.65 (1.23-60.6)	**.03**
**Position**				
Supine	Ref.			
Semi-lateral/lateral	1.16 (0.41-3.33)	.78		
Prone	0.63 (0.07-5.90)	.69		
**Entity**				
Cerebrovascular	Ref.		Ref.	
Tumor	6.18 (0.80-47.6)	**.03**	3.41 (0.35-33.1)	.29
Others	3.50 (0.35-35.4)	.29	1.68 (0.09-30.8)	.73
**Operating duration**	1.00 (0.99-1.01)	.09		
**ClassIntra grade**				
0	Ref.		Ref.	
I	0.78 (0.14-4.36)	.77	0.69 (0.12-4.08)	.69
II	21.7 (5.88-79.9)	**<.01**	26.3 (5.66-121.9)	**<.01**
III	234.0 (23.6-2321.6)	**<.01**	243.2 (20.4-2904.9)	**<.01**

Supplementary table 4: Uni- and multivariate analysis identifying risk factors for a Clavien-Dindo grade >II after elective craniotomy. The analysis included 209 patients, of whom 53 patients suffered from postoperative complications graded with a Clavien-Dindo grade >II.

**Feature**	**Univariate**		**Multivariate**	
	**OR (95%-CI)**	**p-value**	**OR (95%-CI)**	***P* value**
**Age**	1.03 (1.00-1.05)	**.02**	1.02 (0.99-1.04)	.13
**Gender**				
Male	Ref.			
Female	1.25 (0.65-2.37)	.51		
**ASA**				
I / II	Ref.		Ref.	
III / IV	2.42 (1.28-4.56)	**<.01**	1.36 (0.59-3.12)	.48
**BMI**	1.03 (0.98-1.09)	.23		
**Anticoagulation**				
None	Ref.		Ref.	
Paused	2.30 (0.84-6.33)	.11	1.25 (0.34-4.64)	.74
Under medication	6.33 (1.76-22.7)	**<.01**	2.65 (0.50-13.9)	.25
**Charlson Comorbidity Index**	1.22 (1.09-1.35)	**<.01**	1.07 (0.88-1.28)	.51
**Karnofsky**	0.96 (0.94-0.98)	**<.01**	1.01 (0.96-1.06)	.69
**GCS**	0.17 (0.04-0.87)	**.03**	0.27 (0.04-1.68)	.27
**mRS**	1.95 (1.39-2.73)	**<.01**	1.23 (0.38-3.97)	.73
**NIHSS**	1.19 (1.08-1.31)	**<.01**	1.06 (0.76-1.48)	.74
**NANO**	1.38 (1.16-1.65)	**<.01**	1.08 (0.54-2.15)	.84
**Location**				
Supratentorial	Ref.			
Infratentorial	0.61 (0.24-1.57)	.31		
**Side**				
Left	Ref.			
Right	0.99 (0.52-1.91)	.98		
Midline	0.67 (0.20-2.18)	.67		
**Position**				
Supine	Ref.			
Semi-lateral/lateral	1.04 (0.49-2.22)	.91		
Prone	0.22 (0.03-1.85)	.16		
**Entity**				
Cerebrovascular	Ref.			
Tumor	2.07 (0.80-5.35)	.13		
Others	1.11 (0.32-3.86)	.87		
**Operating duration**	1.00 (0.99-1.01)	.13		
**ClassIntra grade**				
0	Ref.		Ref.	
I	2.19 (1.02-4.71)	.05	2.19 (0.94-5.13)	.07
II	7.44 (2.74-20.2)	**<.01**	5.93 (1.93-18.3)	**<.01**
III	24.8 (4.79-128.2)	**<.01**	24.3 (4.13-142.5)	**<.01**

Supplementary table 5: Characteristics and outcome in patients who underwent elective brain tumor surgery.

Feature	All	Grade 0	Grade I	Grade II	Grade III	*P* value
No., n (%)	118 (100.0)	57 (48.3)	40 (33.9)	15 (12.7)	6 (5.1)	
Age [years], mean (SD)	56.9 (13.9)	55.0 (14.2)	57.8 (14.1)	60.5 (14.6)	59.5 (7.2)	.49
Gender, n (%)						
Female	71 (60.2)	38 (53.5)	23 (32.4)	6 (8.5)	4 (5.6)	.29
Male	47 (39.8)	19 (40.4)	17 (36.2)	9 (19.1)	2 (4.3)	
ASA, n (%)						
I	3 (2.5)	2 (66.7)	1 (33.3)	0 (0.0)	0 (0.0)	.91
II	65 (55.1)	34 (52.3)	21 (32.3)	7 (10.8)	3 (4.6)	
III	49 (41.5)	21 (42.9)	17 (34.7)	8 (16.3)	3 (6.1)	
IV	1 (0.8)	0 (0.0)	1 (100.0)	0 (0.0)	0 (0.0)	
BMI, mean (SD)	26.8 (5.9)	26.1 (6.1)	27.4 (6.1)	29.0 (4.2)	22.5 (4.1)	.11
Charlson Comorbidity index, mean (SD)	3.7 (3.1)	3.5 (3.1)	3.5 (2.8)	4.3 (3.3)	5.5 (4.3)	.36
Location, n (%)						
Frontal	53 (44.9)	22 (41.5)	19 (35.8)	8 (15.1)	4 (7.5)	.46
Parietal	40 (33.9)	15 (37.5)	14 (35.0)	7 (17.5)	4 (10.0)	.14
Temporal	25 (21.2)	15 (60.0)	7 (28.0)	3 (12.0)	0 (0.0)	.42
Occipital	23 (19.5)	9 (39.1)	8 (34.8)	5 (21.7)	1 (4.3)	.50
Infratentorial	14 (11.9)	4 (28.6)	7 (50.0)	2 (14.3)	1 (7.1)	.45
Eloquent	59 (50.0)	23 (39.0)	21 (35.6)	9 (15.3)	6 (10.2)	**.03**
Side, n (%)						
Left	52 (44.1)	27 (51.9)	15 (28.8)	8 (15.4)	2 (3.8)	.28
Right	53(44.9)	26 (49.1)	21 (39.6)	4 (7.5)	2 (3.8)	
Midline	13 (11.0)	4 (30.8)	4 (30.8)	3 (23.1)	2 (15.4)	
Pathology, n (%)						
Metastasis	26 (22.0)	13 (50.0)	8 (30.8)	3 (11.5)	2 (7.7)	.92
Meningeoma	47 (39.8)	22 (46.8)	15 (31.9)	8 (17.0)	2 (4.3)	
Glioma	45 (38.1)	22 (48.9)	17 (37.8)	4 (8.9)	2 (4.4)	
Largest diameter [cm], mean (SD)	3.8 (1.6)	3.9 (1.7)	3.8 (1.4)	3.6 (1.4)	3.3 (1.3)	.61
Operating duration [min], mean (SD)	184.4 (84.4)	164.3 (67.5)	193.5 (82.7)	216.9 (81.8)	234.0 (179.5)	**.04**
Unscheduled CT/MRI scan, n (%)	25 (21.2)	7 (12.3)	7 (17.5)	6 (40.0)	6 (83.3)	**<.01**
Reoperation, n (%)	6 (5.1)	1 (1.8)	2 (5.0)	2 (13.3)	1 (16.7)	.17
Highest Clavien-Dindo grade, n (%)						
0	67 (56.8)	39 (68.4)	21 (52.5)	7 (46.8)	0 (0.0)	**<.01**
I	16 (13.6)	8 (14.0)	6 (15.0)	1 (6.7)	1 (16.7)	
II	24 (20.3)	9 (15.8)	10 (25.0)	4 (26.7)	1 (16.7)	
IIIa	2 (1.7)	0 (0.0)	1 (2.5)	1 (6.7)	0 (0.0)	
IIIb	6 (5.1)	1 (1.8)	2 (5.0)	1 (6.7)	2 (33.3)	
IVa	2 (1.7)	0 (0.0)	0 (0.0)	1 (6.7)	1 (16.7)	
IVb	0 (0.0)	0 (0.0)	0 (0.0)	0 (0.0)	0 (0.0)	
V	1 (0.8)	0 (0.0)	0 (0.0)	0 (0.0)	1	
Comprehensive Complication Index, mean (SD)	10.8 (17.4)	5.4 (9.4)	9.6 (11.9)	18.0 (21.9)	52.4 (31.9)	**<.01**
Karnofsky worsening, n (%)	19 (16.1)	5 (8.8)	1 (2.5)	7 (46.7)	6 (100.0)	**<.01**
Glasgow coma scale worsening, n (%)	7 (5.9)	1 (1.8)	0 (0.0)	2 (13.3)	4 (66.7)	**<.01**
Modified Rankin scale worsening, n (%)	19 (16.1)	4 (7.0)	2 (5.0)	7 (46.7)	6 (100.0)	**<.01**
NIHSS worsening, n (%)	19 (16.1)	4 (7.0)	2 (5.0)	7 (46.7)	6 (100.0)	**<.01**
NANO worsening, n (%)	20 (16.9)	5 (8.8)	3 (7.5)	6 (40.0)	6 (100.0)	**<.01**
Length of ICU stay, mean (SD)	1.6 (3.0)	1.1 (0.5)	1.1 (0.6)	3.2 (6.7)	5.2 (7.3)	**<.01**
Length of hospital stay, mean (SD)	7.5 (8.2)	6.1 (2.5)	6.9 (4.9)	12.5 (20.0)	11.7 (6.7)	**.03**

Supplementary table 6: Characteristics and outcome in patients who underwent elective craniotomy for cerebral metastasis.

Feature	All	Grade 0	Grade I	Grade ≥ II	*P* value
No., n (%)	26 (100.0)	13 (50.0)	8 (30.8)	5 (19.2)	
Age [years], mean (SD)	59.2 (12.4)	57.7 (12.1)	59.4 (15.9)	62.6 (7.6)	.77
Gender, n (%)					
Female	20 (76.9)	12 (60.0)	6 (30.0)	2 (10.0)	.06
Male	6 (23.1)	1 (16.7)	2 (33.3)	3 (50.0)	
ASA, n (%)					
II	11 (42.3)	7 (63.6)	4 (36.4)	0 (0.0)	.10
III	15 (57.7)	6 (40.0)	4 (26.7)	5 (33.3)	
BMI, mean (SD)	25.3 (5.8)	25.2 (6.6)	24.2 (4.5)	27.2 (6.1)	.68
Charlson Comorbidity index, mean (SD)	6.9 (3.4)	7.2 (2.8)	4.4 (3.5)	10.0 (1.2)	**<.01**
Location, n (%)					
Frontal	8 (30.8)	3 (37.5)	3 (37.5)	2 (25.0)	.69
Parietal	8 (30.8)	4 (50.0)	2 (25.0)	2 (25.0)	.85
Temporal	2 (7.7)	0 (0.0)	1 (50.0)	1 (50.0)	.30
Occipital	8 (30.8)	5 (62.5)	0 (0.0)	3 (37.5)	.06
Infratentorial	9 (34.6)	3 (33.3)	4 (44.4)	2 (22.2)	.44
Eloquent	20 (76.9)	8 (40.0)	7 (35.0)	5 (25.0)	.15
Side, n (%)					
Left	15 (57.7)	9 (60.0)	2 (13.3)	2 (26.7)	.09
Right	9 (34.6)	4 (44.4)	5 (55.6)	0 (0.0)	
Midline	2 (7.7)	0 (0.0)	1 (50.0)	1 (50.0)	
Primary tumor, n (%)					
Lung carcinoma	13 (50.0)	6 (46.2)	6 (46.2)	1 (7.7)	.27
Renal cell carcinoma	1 (3.8)	0 (0.0)	0 (0.0)	1 (100.0)	
Breast cancer	4 (15.4)	3 (75.0)	1 (25.0)	0 (0.0)	
Malignant melanoma	3 (11.5)	1 (33.3)	0 (0.0)	2 (66.7)	
GI cancer	3 (11.5)	1 (33.3)	1 (33.3)	1 (33.3)	
Ewing sarcoma	1 (3.8)	1 (100.0)	0 (0.0)	0 (0.0)	
Ovarial cancer	1 (3.8)	1 (100.0)	0 (0.0)	0 (0.0)	
Largest diameter [cm], mean (SD)	3.5 (1.5)	3.7 (1.8)	3.4 (1.5)	3.2 (1.1)	.79
No. of resected metastases					
1	24 (92.3)	12 (50.0)	8 (33.3)	1 (16.7)	.42
≥ 2	2 (7.7)	1 (50.0)	0 (0.0)	1 (50.0)	
Recurrent surgery, n (%)	4 (15.4)	3 (75.0)	0 (0.0)	1 (25.0)	.35
RPA score					
1	8 (30.8)	4 (50.0)	4 (50.0)	0 (0.0)	.34
2	16 (61.5)	8 (50.0)	4 (25.0)	4 (25.0)	
3	2 (7.7)	1 (50.0)	0 (0.0)	1 (50.0)	
GPA score					
0.0-1.0	4 (15.4)	1 (25.0)	1 (25.0)	2 (50.0)	.23
1.5-2.0	8 (30.8)	5 (62.5)	1 (12.5)	2 (25.0)	
≥2.5	12 (46.2)	5 (41.7)	6 (50.0)	1 (8.3)	
Operating duration [min], mean (SD)	156.1 (56.5)	146.1 (56.3)	157.1 (35.3)	180.4 (84.6)	
Unscheduled CT/MRI scan, n (%)	6 (23.1)	2 (15.4)	1 (12.5)	3 (60.0)	**.04**
Highest Clavien-Dindo grade, n (%)					
≤ II	23 (88.5)	13 (100.0)	8 (100.0)	2 (40.0)	**<.01**
≥ IIIa	3 (11.5)	0 (0.0)	0 (0.0)	3 (60.0)	
Comprehensive Complication Index, mean (SD)	15.2 (23.0)	7.8 (11.1)	10.8 (13.7)	41.4 (39.2)	**.01**
Karnofsky worsening, n (%)	4 (15.4)	1 (7.7)	0 (0.0)	3 (60.0)	**<.01**
Glasgow coma scale worsening, n (%)	3 (11.5)	1 (7.7)	0 (0.0)	2 (40.0)	.07
Modified Rankin scale worsening, n (%)	4 (15.4)	1 (7.7)	0 (0.0)	3 (60.0)	**<.01**
NIHSS worsening, n (%)	4 (15.4)	1 (7.7)	0 (0.0)	3 (60.0)	**<.01**
NANO worsening, n (%)	3 (11.5)	1 (7.7)	0 (0.0)	2 (40.0)	.07
Length of ICU stay, mean (SD)	1.2 (0.6)	1.2 (0.6)	1.0 (0.0)	1.8 (0.8)	**.04**
Length of hospital stay, mean (SD)	6.7 (3.2)	6.4 (3.3)	7.4 (2.9)	6.4 (3.6)	.78

Supplementary table 7: Characteristics and outcome in patients who underwent elective craniotomy for meningioma.

Feature	All	Grade 0	Grade I	Grade ≥ II	*P* value
No., n (%)	47 (100.0)	22 (46.8)	15 (31.9)	10 (21.3)	
Age [years], mean (SD)	57.9 (12.7)	56.2 (13.5)	59.8 (12.6)	59.1 (11.9)	.68
Gender, n (%)					
Female	33 (70.2)	18 (54.5)	9 (27.3)	6 (18.2)	.26
Male	14 (29.8)	4 (28.6)	6 (42.9)	4 (28.6)	
ASA, n (%)					
I	2 (4.3)	1 (50.0)	1 (50.0)	0 (0.0)	.72
II	28 (59.6)	14 (50.0)	7 (25.0)	7 (25.0)	
III	16 (34.0)	7 (43.8)	6 (37.5)	3 (18.8)	
IV	1 (2.1)	0 (0.0)	1 (100.0)	0 (0.0)	
BMI, mean (SD)	28.9 (6.9)	27.4 (7.4)	30.8 (6.9)	29.1 (5.2)	.36
Charlson Comorbidity index, mean (SD)	2.7 (2.1)	2.2 (1.9)	3.5 (2.5)	2.4 (1.7)	.19
Location, n (%)					
Skull base	16 (34.0)	6 (37.5)	6 (37.5)	4 (25.0)	.74
Convexity	12 (25.5)	7 (58.3)	3 (25.0)	2 (16.7)	
Parasagittal	7 (14.9)	4 (57.1)	3 (42.9)	0 (0.0)	
Tentorial	5 (10.6)	1 (20.0)	2 (40.0)	2 (40.0)	
Cerebellar	1 (2.1)	1 (100.0)	0 (0.0)	0 (0.0)	
Falx	6 (12.8)	3 (50.0)	1 (16.7)	2 (33.3)	
Sinus infiltration, n (%)	9 (19.1)	4 (44.4)	4 (44.4)	1 (11.1)	.58
Side, n (%)					
Left	19 (40.4)	11 (57.9)	4 (21.1)	4 (21.1)	.11
Right	20 (42.6)	9 (45.0)	9 (45.0)	2 (10.0)	
Midline	8 (17.0)	2 (25.0)	2 (25.0)	4 (50.0)	
Largest diameter [cm], mean (SD)	3.9 (1.4)	4.0 (1.5)	3.6 (1.2)	4.0 (1.4)	.56
Simpson grade, n (%)					
1	9 (20.5)	6 (66.7)	2 (22.2)	1 (11.1)	.42
2	22 (50.0)	10 (45.5)	6 (27.3)	6 (27.3)	
3	5 (11.4)	2 (40.0)	3 (60.0)	0 (0.0)	
4	8 (25.0)	2 (25.0)	3 (37.5)	3 (37.5)	
Recurrent surgery, n (%)	4 (8.5)	3 (13.6)	1 (6.7)	0 (0.0)	.42
WHO grade, n (%)					
1	37 (80.4)	16 (76.2)	13 (35.1)	8 (21.6)	.82
2	8 (17.4)	4 (50.0)	2 (25.0)	2 (25.0)	
Unknown	1 (2.2)	1 (100.0)	0 (0.0)	0 (0.0)	
Operating duration [min], mean (SD)	224.4 (107.7)	182.9 (82.9)	254.5 (100.9)	270.4 (139.2)	**.04**
Reoperation, n (%)	5 (10.6)	1 (4.5)	2 (13.3)	2 (20.0)	.39
Unscheduled CT/MRI scan, n (%)	15 (31.9)	4 (18.2)	5 (33.3)	6 (60.0)	.06
Highest Clavien-Dindo grade, n (%)					
≤ II	41 (87.2)	21 (51.2)	12 (29.3)	8 (19.5)	.29
≥ IIIa	6 (12.8)	1 (16.7)	3 (50.0)	2 (33.3)	
Comprehensive Complication Index, mean (SD)	12.6 (15.9)	6.6 (10.5)	14.3 (13.1)	23.4 (23.5)	**.02**
Karnofsky worsening, n (%)	11 (23.4)	3 (13.6)	1 (6.7)	7 (70.0)	**<.01**
Glasgow coma scale worsening, n (%)	2 (4.3)	0 (0.0)	0 (0.0)	2 (20.0)	**<.02**
Modified Rankin scale worsening, n (%)	11 (23.4)	2 (9.1)	2 (13.3)	7 (70.0)	**<.01**
NIHSS worsening, n (%)	11 (23.4)	2 (9.1)	2 (13.3)	7 (70.0)	**<.01**
NANO worsening, n (%)	12 (25.5)	2 (9.1)	3 (20.0)	7 (70.0)	**<.01**
Length of ICU stay, mean (SD)	1.7 (2.9)	1.1 (0.6)	1.4 (0.8)	3.5 (6.0)	.09
Length of hospital stay, mean (SD)	6.1 (2.5)	8.5 (7.0)	9.8 (4.9)	7.7 (4.9)	.11

Supplementary table 8: Characteristics and outcome in patients who underwent elective craniotomy for glioma.

Feature	All	Grade 0	Grade I	Grade ≥ II	*P* value
No., n (%)	45 (100.0)	22 (48.9)	17 (37.8)	6 (13.3)	
Age [years], mean (SD)	54.4 (15.9)	52.2 (16.1)	55.4 (14.9)	60.2 (18.5)	.54
Gender, n (%)					
Female	18 (40.0)	8 (44.4)	8 (44.4)	2 (11.1)	.75
Male	27 (60.0)	14 (51.9)	9 (33.3)	4 (14.8)	
ASA, n (%)					
I	1 (2.2)	1 (100.0)	0 (0.0)	0 (0.0)	.85
II	26 (57.8)	13 (50.0)	10 (38.5)	3 (11.5)	
III	18 (40.0)	8 (44.4)	7 (38.9)	3 (16.7)	
BMI, mean (SD)	25.5 (4.1)	25.6 (4.5)	25.7 (4.1)	25.1 (3.0)	.95
Charlson Comorbidity index, mean (SD)	2.9 (2.5)	2.5 (2.5)	3.1 (2.6)	4.0 (2.2)	.41
Location, n (%)					
Frontal	18 (40.0)	7 (38.9)	7 (38.9)	4 (22.2)	.30
Parietal	17 (37.8)	5 (29.4)	7 (41.2)	5 (29.4)	**.02**
Temporal	15 (33.3)	10 (66.7)	4 (26.7)	1 (6.7)	.23
Occipital	7 (15.6)	3 (42.9)	4 (57.1)	0 (0.0)	.37
Eloquent	23 (51.1)	10 (43.5)	8 (34.8)	5 (21.7)	.24
Side, n (%)					
Left	18 (40.0)	7 (38.9)	9 (50.0)	2 (11.1)	.63
Right	24 (53.3)	13 (54.2)	7 (29.2)	4 (16.7)	
Midline	3 (6.7)	2 (66.7)	1 (33.3)	0 (0.0)	
Largest diameter [cm], mean (SD)	3.9 (1.7)	4.1 (1.9)	4.2 (1.4)	2.8 (1.4)	.24
Pathology, n (%)					
Glioblastoma	31 (68.9)	16 (51.6)	11 (35.5)	4 (12.9)	.41
Astrocytoma	5 (11.1)	2 (40.0)	2 (40.0)	1 (20.0)	
Oligodendroglioma	6 (13.3)	3 (50.0)	3 (50.0)	0 (0.0)	
H3K27-mutated	1 (2.2)	0 (0.0)	0 (0.0)	1 (100.0)	
Pleomorphic xanthoastrocytoma	2 (4.4)	1 (50.0)	1 (50.0)	0 (0.0)	
Recurrent surgery, n (%)	16 (35.6)	7 (43.8)	5 (31.3)	4 (25.0)	.23
WHO grade, n (%)					
2	6 (13.3)	2 (33.3)	4 (66.7)	0 (0.0)	.57
3	7 (15.6)	4 (57.1)	2 (28.6)	1 (14.3)	
4	32 (71.1)	16 (50.0)	11 (34.4)	5 (15.6)	
IDH status, n (%)					
Wild type	33 (73.3)	16 (48.5)	12 (36.4)	5 (15.2)	.83
Mutated	12 (26.7)	6 (50.0)	5 (41.7)	1 (8.3)	
MGMT promotor, n (%)					
Non-methylated	20 (46.5)	9 (45.0)	8 (40.0)	3 (15.0)	.89
Methylated	23 (53.5)	12 (52.2)	8 (34.8)	3 (13.0)	
Extent of resection, n (%)					
GTR	13 (28.9)	9 (69.2)	3 (23.1)	1 (7.7)	.15
Near GTR	21 (46.7)	8 (38.1)	8 (38.1)	5 (23.8)	
Partial resection or biopsy	11 (24.4)	5 (45.5)	6 (54.5)	0 (0.0)	
Operating duration [min], mean (SD)	158.9 (46.5)	156.3 (53.4)	156.7 (40.1)	175.2 (39.1)	.67
Reoperation, n (%)	1 (2.2)	0 (0.0)	0 (0.0)	1 (16.7)	**.04**
Unscheduled CT/MRI scan, n (%)	4 (8.9)	1 (4.5)	1 (5.9)	2 (33.3)	.08
Highest Clavien-Dindo grade, n (%)					
≤ II	43 (95.6)	22 (100.0)	17 (100.0)	4 (66.7)	**<.01**
≥ IIIa	2 (4.4)	0 (0.0)	0 (0.0)	2 (33.3)	
Comprehensive Complication Index, mean (SD)	6.3 (14.2)	2.7 (6.4)	4.8 (8.5)	23.9 (30.4)	**<.01**
Karnofsky worsening, n (%)	4 (8.9)	1 (4.5)	0 (0.0)	3 (50.0)	**<.01**
Glasgow coma scale worsening, n (%)	2 (4.4)	0 (0.0)	0 (0.0)	2 (33.3)	**<.01**
Modified Rankin scale worsening, n (%)	4 (8.9)	1 (4.5)	0 (0.0)	3 (50.0)	**<.01**
NIHSS worsening, n (%)	4 (8.9)	1 (4.5)	0 (0.0)	3 (50.0)	**<.01**
NANO worsening, n (%)	5 (11.1)	2 (9.1)	0 (0.0)	3 (50.0)	**<.01**
Length of ICU stay, mean (SD)	1.6 (3.9)	1.0 (0.0)	0.94 (0.2)	5.8 (10.4)	**.01**
Length of hospital stay, mean (SD)	7.7 (11.9)	5.9 (2.0)	5.2 (2.2)	21.3 (31.2)	**<.01**

Supplementary table 9: Characteristics and outcome in patients who underwent elective microsurgical clipping of an unruptured intracranial aneurysm.

Feature	All	Grade 0	Grade I	Grade II	*P* value
No., n (%)	31	16 (51.6)	12 (38.7)	3 (9.7)	
Age [years], mean (SD)	57.7 (11.5)	54.3 (11.9)	59.7 (9.6)	68.3 (11.7)	.11
Gender, n (%)					
Female	22 (71.0)	10 (45.5)	9 (40.9)	3 (13.6)	.39
Male	9 (29.0)	6 (66.7)	3 (33.3)	0 (0.0)	
ASA, n (%)					
II	18 (58.1)	10 (55.6)	7 (38.9)	1 (5.6)	.64
III	6 (46.2)	6 (46.2)	5 (38.5)	2 (15.4)	
BMI, mean (SD)	27.5 (5.1)	27.7 (5.2)	28.3 (4.9)	23.1 (4.0)	.28
Charlson Comorbidity index, mean (SD)	2.4 (1.8)	2.6 (2.2)	2.0 (1.0)	3.0 (2.0)	.56
Location, n (%)					
ACOM	4 (12.9)	1 (25.0)	3 (75.0)	0 (0.0)	.33
ACA-A1	2 (6.5)	0 (0.0)	2 (100.0)	0 (0.0)	
ACA-A3	2 (6.5)	1 (50.0)	1 (50.0)	0 (0.0)	
MCA-M2	1 (3.2)	1 (100.0)	0 (0.0)	0 (0.0)	
MCA-M3	17 (54.8)	9 (52.9)	5 (29.4)	3 (17.6)	
ICA pars cavernosus	4 (12.9)	4 (100.0)	0 (0.0)	0 (0.0)	
Basilar artery	1 (3.2)	0 (0.0)	1 (100.0)	0 (0.0)	
PHASES score, n (%)					
≤ 5	23 (74.2)	14 (87.5)	7 (58.3)	2 (66.6)	.11
> 5	8 (25.8)	2 (12.5)	5 (41.7)	1 (33.4)	
Largest diameter [mm], mean (SD)	6.3 (4.4)	4.8 (1.7)	7.9 (6.0)	9.0 (6.9)	.13
Neck diameter [mm], mean (SD)	3.5 (1.8)	2.9 (1.1)	4.7 (2.6)	3.3 (1.5)	.10
Aneurysm calcification, n (%)					
No	13 (48.1)	7 (53.8)	4 (30.8)	2 (15.4)	.79
Yes	14 (51.9)	8 (57.1)	5 (35.7)	1 (7.1)	
Aneurysm morphology, n (%)					
Regular	18 (58.1)	13 (72.2)	3 (16.7)	2 (11.1)	**.01**
Irregular	13 (41.9)	3 (23.1)	9 (69.2)	1 (7.7)	
No. of clips needed, n (%)					
1	25 (80.6)	16 (64.0)	7 (28.0)	2 (8.0)	**.02**
> 1	6 (19.4)	0 (0.0)	5 (83.3)	1 (16.7)	
Temporary vessel occlusion, n (%)					
No	20 (64.5)	13 (65.0)	5 (25.0)	2 (10.0)	.09
Yes	11 (35.5)	3 (27.3)	7 (63.6)	1 (9.1)	
Clip repositioning, n (%)					
No	29 (64.5)	13 (65.0)	5 (25.0)	2 (10.0)	.09
Yes	11 (35.5)	3 (27.3)	7 (63.6)	1 (9.1)	
Postoperative remnant, n (%)					
No	25 (80.6)	15 (60.0)	8 (32.0)	2 (8.0)	.16
Yes	6 (19.4)	1 (16.7)	4 (66.7)	1 (16.7)	
Operating duration [min], mean (SD)	141.9 (48.7)	127.6 (41.8)	159.8 (58.1)	147.3 (15.7)	.22
Unscheduled CT/MRI scan, n (%)	3 (9.7)	1 (6.3)	2 (16.7)	0 (0.0)	.55
Highest Clavien-Dindo grade, n (%)					
0	20 (64.5)	13 (81.3)	6 (50.0)	1 (33.3)	**.04**
I	6 (19.4)	2 (12.5)	4 (33.3)	0 (0.0)	
II	5 (16.1)	1 (6.3)	2 (16.7)	2 (66.7)	
Comprehensive Complication Index, mean (SD)	6.3 (10.5)	3.6 (9.4)	7.2 (9.8)	16.8 (15.2)	**.02**
Karnofsky worsening, n (%)	1 (3.2)	0 (0.0)	1 (8.3)	0 (0.0)	.44
Glasgow coma scale worsening, n (%)	0 (0.0)	0 (0.0)	0 (0.0)	0 (0.0)	n.a.
Modified Rankin scale worsening, n (%)	1 (3.2)	0 (0.0)	1 (8.3)	0 (0.0)	.44
NIHSS worsening, n (%)	1 (3.2)	0 (0.0)	1 (8.3)	0 (0.0)	.44
NANO worsening, n (%)	1 (3.2)	0 (0.0)	1 (8.3)	0 (0.0)	.44
Length of ICU stay, mean (SD)	1.23 (0.7)	1.19 (0.8)	1.25 (0.6)	1.33 (0.6)	.93
Length of hospital stay, mean (SD)	5.4 (2.1)	5.0 (1.4)	5.7 (3.1)	6.0 (1.0)	.63

Supplementary table 10: Characteristics and outcome in patients who underwent emergent craniotomy.

Feature	All	Grade 0	Grade I	Grade II	Grade III	Grade IV	*P* value
No., n (%)	32	11 (34.4)	3 (9.4)	8 (25.0)	5 (15.6)	5 (15.6)	
Age [years], mean (SD)	63.6 (16.6)	65.4 (15.8)	59.3 (22.5)	67.4 (15.2)	54.8 (12.5)	64.8 (23.3)	.73
Gender, n (%)							
Female	14 (43.8)	6 (42.9)	2 (14.3)	3 (21.4)	1 (7.1)	2 (14.3)	.65
Male	18 (56.3)	5 (27.8)	1 (5.6)	5 (27.8)	4 (22.2)	3 (16.7)	
ASA, n (%)							
II	2 (6.3)	0 (0.0)	0 (0.0)	2 (100.0)	0 (0.0)	0 (0.0)	.15
III	6 (18.8)	2 (33.3)	2 (33.3)	1 (16.7)	0 (0.0)	1 (16.7)	
IV	7 (21.9)	3 (42.9)	1 (14.3)	1 (14.3)	0 (0.0)	2 (28.6)	
V	17 (53.1)	6 (35.3)	0 (0.0)	4 (23.5)	5 (29.4)	2 (11.8)	
BMI, mean (SD)	25.9 (5.3)	23.5 (3.8)	25.9 (4.1)	27.9 (4.9)	27.6 (9.3)	26.3 (1.9)	.43
Anticoagulation, n (%)							
None	21 (65.6)	8 (38.1)	2 (9.5)	3 (14.3)	5 (23.8)	3 (14.3)	.22
Paused	0 (0.0)	0 (0.0)	0 (0.0)	0 (0.0)	0 (0.0)	0 (0.0)	
Under medication	11 (34.4)	3 (27.3)	1 (9.1)	5 (45.5)	0 (0.0)	2 (18.2)	
Charlson Comorbidity index, mean (SD)	5.5 (3.5)	4.6 (2.3)	5.3 (2.9)	5.1 (2.4)	6.8 (5.5)	5.4 (5.5)	.85
Karnofsky, mean (SD)	48.7 (14.6)	55.0 (10.0)	45.0 (21.2)	50.0 (18.7)	35.0 (7.0)	50.0 (14.1)	.67
Glasgow coma scale, mean (SD)	10.5 (3.2)	12.0 (2.2)	10.5 (2.1)	10.6 (4.1)	8.0 (2.8)	9.5 (4.9)	.73
Modified Rankin scale, mean (SD)	3.5 (0.7)	3.8 (1.5)	4.0 (1.4)	2.8 (1.8)	4.5 (0.7)	3.5 (0.8)	.69
NIHSS, mean (SD)	10.5 (5.4)	6.3 (2.9)	13.5 (6.4)	9.6 (4.4)	18.0 (5.7)	10.5 (4.9)	.10
NANO, mean (SD)	8.4 (3.7)	6.3 (2.4)	11.5 (3.5)	7.4 (3.6)	15.0 (5.1)	8.5 (3.5)	.17
Ventilated admission, n (%)	17 (53.1)	7 (41.2)	1 (5.9)	3 (17.6)	3 (17.6)	3 (17.6)	.75
Location, n (%)							
Supratentorial	30 (93.8)	10 (33.3)	3 (10.0)	7 (23.3)	5 (16.7)	5 (16.7)	.82
Infratentorial	2 (6.2)	1 (50.0)	0 (0.0)	1 (50.0)	0 (0.0)	0 (0.0)	
Side, n (%)							
Left	10 (31.3)	4 (40.0)	2 (20.0)	3 (30.0)	0 (0.0)	1 (10.0)	.33
Right	22 (68.7)	7 (31.8)	1 (4.5)	5 (22.7)	5 (22.7)	4 (18.2)	
Procedure, n (%)							
Abscess	3 (9.4)	2 (66.7)	0 (0.0)	0 (0.0)	0 (0.0)	1 (33.3)	.92
Aneurysm	7 (21.9)	2 (28.6)	1 (14.3)	2 (28.6)	1 (14.3)	1 (14.3)	
Acute subdural hematoma	8 (25.0)	1 (12.5)	1 (12.5)	3 (37.5)	1 (12.5)	2 (25.0)	
Hemicraniectomy	7 (21.9)	3 (42.9)	1 (14.3)	1 (14.3)	2 (28.6)	0 (0.0)	
Intraparenchymal bleeding	7 (21.9)	3 (42.9)	0 (0.0)	2 (28.6)	1 (14.3)	1 (14.3)	
Operating duration [min], mean (SD)	103.5 (46.9)	100.9 (42.3)	90.7 (42.6)	109.6 (61.6)	93.8 (39.2)	116.0 (53.2)	.93
Unscheduled CT/MRI scan, n (%)	17 (53.1)	2 (18.2)	1 (33.3)	6 (75.0)	4 (80.0)	4 (80.0)	**.04**
Reoperation, n (%)	8 (25.0)	0 (0.0)	0 (0.0)	5 (62.5)	1 (20.0)	2 (40.0)	**.02**
Highest Clavien-Dindo grade, n (%)							
0	3 (9.4)	3 (27.3)	0 (0.0)	0 (0.0)	0 (0.0)	0 (0.0)	**.03**
I	1 (3.1)	0 (0.0)	1 (33.3)	0 (0.0)	0 (0.0)	0 (0.0)	
II	7 (21.9)	3 (27.3)	1 (33.3)	3 (37.5)	0 (0.0)	0 (0.0)	
IIIa	2 (6.3)	2 (18.2)	0 (0.0)	0 (0.0)	0 (0.0)	0 (0.0)	
IIIb	5 (15.6)	0 (0.0)	0 (0.0)	3 (37.5)	1 (20.0)	1 (20.0)	
IVa	2 (6.3)	2 (18.2)	0 (0.0)	0 (0.0)	0 (0.0)	0 (0.0)	
IVb	0 (0.0)	0 (0.0)	0 (0.0)	0 (0.0)	0 (0.0)	0 (0.0)	
V	12 (37.5)	1 (9.1)	1 (33.3)	2 (25.0)	4 (80.0)	4 (80.0)	
Comprehensive Complication Index, mean (SD)	58.7 (37.2)	31.3 (30.0)	43.2 (49.6)	61.4 (29.1)	93.8 (13.8)	88.9 (24.7)	**<.01**
Karnofsky, mean (SD)	51.6 (20.9)	56.7 (24.5)	60.0 (14.1)	45.0 (18.7)	50.0 (14.9)	30.0 (15.1)	.69
Karnofsky worsening, n (%)	17 (53.1)	3 (27.3)	1 (33.3)	4 (50.0)	4 (80.0)	5 (100.0)	**.02**
Glasgow coma scale, mean (SD)	12.6 (2.2)	13.1 (2.5)	13.5 (2.1)	12.2 (1.7)	13.0 (1.9)	9.0 (1.2)	.49
Glasgow coma scale worsening, n (%)	16 (50.0)	2 (18.2)	1 (33.3)	4 (50.0)	4 (80.0)	5 (100.0)	**<.01**
Modified Rankin scale, mean (SD)	3.0 (1.5)	2.6 (1.5)	3.0 (1.4)	3.3 (1.8)	3.0 (1.3)	5.0 (1.7)	.65
Modified Rankin scale worsening, n (%)	16 (50.0)	2 (18.2)	1 (33.3)	4 (50.0)	4 (80.0)	5 (100.0)	**<.01**
NIHSS, mean (SD)	11.3 (7.4)	9.0 (8.2)	8.0 (5.7)	14.5 (6.9)	12.0 (5.9)	18.0 (7.3)	.57
NIHSS worsening, n (%)	16 (50.0)	2 (18.2)	1 (33.3)	4 (50.0)	4 (80.0)	5 (100.0)	**<.01**
NANO, mean (SD)	7.1 (4.8)	5.6 (5.2)	4.5 (3.5)	9.0 (3.8)	8.0 (3.2)	14.0 (4.2)	.35
NANO worsening, n (%)	17 (53.1)	3 (27.3)	1 (33.3)	4 (50.0)	4 (80.0)	5 (100.0)	**.02**
Length of ICU stay, mean (SD)	9.8 (8.8)	12.4 (10.3)	9.0 (5.2)	10.9 (10.8)	6.2 (6.9)	6.8 (4.4)	.67
Length of hospital stay, mean (SD)	12.8 (10.6)	15.3 (9.3)	11.0 (4.6)	16.8 (15.1)	7.6 (9.9)	**7.0 (4.2)**	.36
